# Video-Assisted Minithoracotomy for Pulmonary Laceration with a Massive Hemothorax

**DOI:** 10.1155/2014/454970

**Published:** 2014-03-13

**Authors:** Hideki Ota, Hideki Kawai, Shuntaro Togashi, Tsubasa Matsuo

**Affiliations:** Department of Thoracic Surgery, Akita Red Cross Hospital, 222-1 Kamikitate, Akita City, Akita 010-1495, Japan

## Abstract

Severe intrathoracic hemorrhage from pulmonary parenchyma is the most serious complication of pulmonary laceration after blunt trauma requiring immediate surgical hemostasis through open thoracotomy. The safety and efficacy of video-assisted thoracoscopic surgery (VATS) techniques for this life-threatening condition have not been fully evaluated yet. We report a case of pulmonary laceration with a massive hemothorax after blunt trauma successfully treated using a combination of muscle-sparing minithoracotomy with VATS techniques (video-assisted minithoracotomy). A 22-year-old man was transferred to our department after a falling accident. A diagnosis of right-sided pneumothorax was made on physical examination and urgent chest decompression was performed with a tube thoracostomy. Chest computed tomographic scan revealed pulmonary laceration with hematoma in the right lung. The pulmonary hematoma extending along segmental pulmonary artery in the helium of the middle lobe ruptured suddenly into the thoracic cavity, resulting in hemorrhagic shock on the fourth day after admission. Emergency right middle lobectomy was performed through video-assisted minithoracotomy. We used two cotton dissectors as a chopstick for achieving compression hemostasis during surgery. The patient recovered satisfactorily. Video-assisted minithoracotomy can be an alternative approach for the treatment of pulmonary lacerations with a massive hemothorax in hemodynamically unstable patients.

## 1. Introduction 

Pulmonary laceration is primarily associated with penetrating injuries and occurs less commonly in blunt trauma with a prevalence of 4.0–6.7% [1, 2]. Severe intrathoracic hemorrhage from pulmonary parenchyma is the most serious complication of pulmonary laceration after blunt trauma requiring immediate surgical hemostasis through open thoracotomy [3, 4]. Video-assisted thoracoscopic surgery (VATS) techniques have been widely applied in trauma patients [5]; however, little is known about their safety and efficacy for achieving surgical hemostasis of hemorrhage from pulmonary laceration in hemodynamically unstable patients. We report a case of pulmonary laceration with a massive hemothorax after blunt trauma successfully treated using a combination of muscle-sparing minithoracotomy with VATS techniques (video-assisted minithoracotomy).

## 2. Case Presentation

A previously healthy 22-year-old man fell from a fifth-floor apartment. He was transferred to our department with unstable cardiopulmonary condition: blood pressure, 72/60 mmHg; pulse rate, 120 beats/min; respiratory rate, 40 breaths/min; SpO_2_, 80% on 10 L/min flow oxygen by reservoir mask. Physical examination revealed diminished breath sounds on the right hemithorax, jugular venous distention, and tracheal deviation to the left side. A diagnosis of right-sided tension pneumothorax was made and an emergency tube thoracostomy was performed. Radiological examinations revealed a right-sided pneumothorax, pulmonary laceration with hematoma in the right lung, traumatic liver injury, and fractures of the right 11th and 12th ribs and the pelvis. Laboratory evaluations indicated a hemoglobin level of 8.6 g/dL; platelet count 65,000/mm^3^/*μ*L; prothrombin time-international normalized ratio 1.53; and fibrin degradation products 307 *μ*g/mL. The patient presented with hemorrhagic shock and acute disseminated intravascular coagulation (DIC). Severe hemorrhage from liver injury and pelvic fracture was controlled successfully by percutaneous transcatheter arterial embolization. He was treated in the intensive care unit under endotracheal intubation and mechanical ventilation, with blood transfusion therapy and anti-DIC treatment. Two days after admission, he presented with atelectasis of the middle lobe of the lung on chest radiography, reduced air leakage, and hemoptysis.

**Figure 1:**
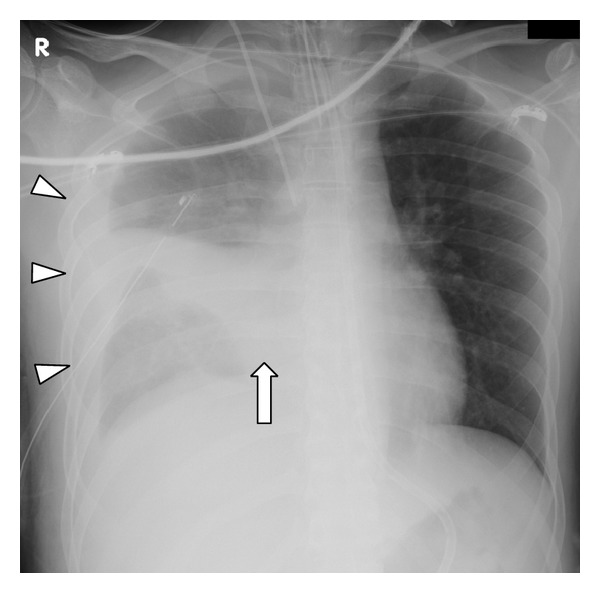
Chest radiograph obtained on the fourth day after admission shows right middle lobe atelectasis (arrow) and right-sided hemothorax (arrowhead).

**Figure 2:**
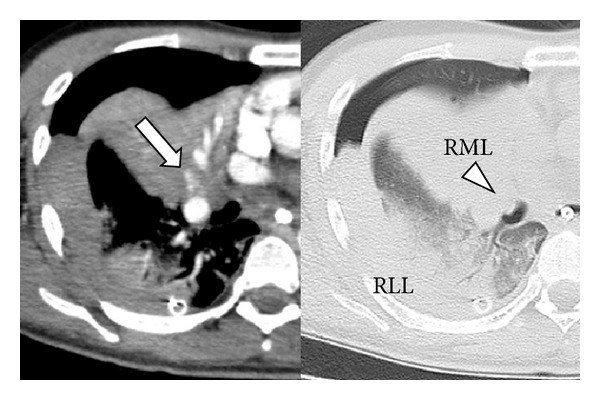
Chest computed tomography reveals extravasation of contrast medium in the right middle lobe (arrow) and compression of the middle lobe bronchus by a pulmonary hematoma (arrowhead). RML: right middle lobe; RLL: right lower lobe.

Four days after admission, he presented with sudden onset of dyspnea and chest pain. Chest radiography showed a right-sided hemothorax (Figure 1). Bronchoscopy demonstrated severe stenosis of the middle lobe bronchus. A blood volume of 1,000 mL was drained through the chest tube over the following 4 hours. His hemoglobin level decreased from 11.3 mg/dL to 8.2 mg/dL. He fell into a state of hemorrhagic shock despite blood transfusion therapy. Chest computed tomography (CT) revealed extravasation of contrast medium in the right middle lobe and compression of the middle lobe bronchus by a pulmonary hematoma (Figure 2). We confirmed a delayed rupture of the pulmonary hematoma into the right thoracic cavity, resulting in hemorrhagic shock

**Figure 3:**
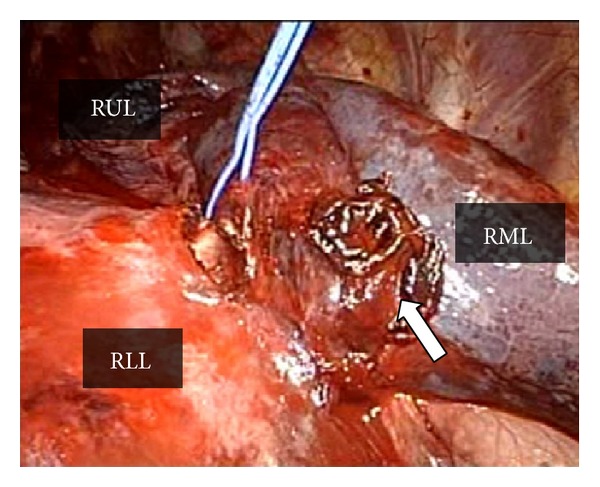
Intraoperative findings show pleural injury with a clotted hematoma in the middle lobe of the right lung (arrow). RUL: right upper lobe; RML: right middle lobe; RLL: right lower lobe.

**Figure 4:**
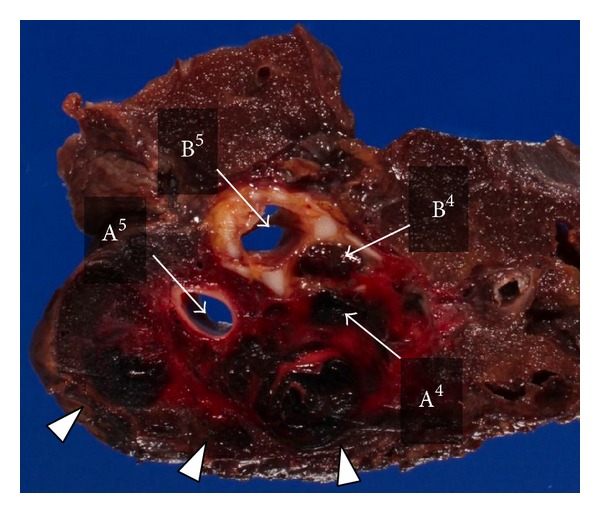
Macroscopic examination of the resected specimen shows disruption of lateral segment artery of the middle lobe of the lung, resulting in a pulmonary hematoma (arrowhead). Lateral segmental bronchus was obstructed by a blood clot. B^4^: lateral segmental bronchus; B^5^: medial segmental bronchus; A^4^: lateral segmental artery; A^5^: medial segmental artery.

Right middle lobectomy was performed through video-assisted minithoracotomy, also known as hybrid surgical approach of minithoracotomy with VATS techniques (hybrid VATS) [6]. A thoracoscope access port was placed in the eighth intercostal space in the anterior axillary line. A muscle-sparing minithoracotomy was performed through a 6 cm transverse skin incision over the middle axillary line in the fifth intercostal space. After evacuating approximately 700 mL of a mixture of fresh and clotted blood, we identified an active bleeding site of ruptured pulmonary hematoma of the middle lobe (Figure 3). Two cotton dissectors (Naruke's thoraco cotton; Kenzmedico Co, Saitama, Japan) were used as a chopstick to obtain compression hemostasis during surgery. After proximal control of interlober pulmonary artery was achieved with nylon tape, individual branches of the pulmonary artery to the right middle lobe were doubly ligated with 2–0 silk suture. Pulmonary vein to the middle lobe was divided by an EndoGIA ultrauniversal tristapler camel 45 mm (Covivien, Menfield, USA). The bronchial stump was closed using interrupted 4–0 monofilament non-absorbable sutures (Prolene; Ethicon, Somerville, NJ, USA). Pathological examination of the resected lung confirmed the disruption of the lateral segment artery of the middle lobe of the lung, leading to pulmonary hematoma (Figure 4). The postoperative course was uneventful, and the patient was weaned from the ventilator on the first postoperative day and recovered from acute DIC on the second postoperative day. He was discharged on the 46th postoperative day after stabilization of the pelvic fracture.

## 3. Discussion 

We herein describe a case of pulmonary laceration with a massive hemothorax successfully treated with video-assisted minithoracotomy. Severe intrathoracic hemorrhage occurred due to the rupture of pulmonary hematoma extending along segmental pulmonary artery in the helium of the middle lobe. Compression hemostasis during surgery was well achieved by VATS techniques using a cotton dissector.

Pulmonary laceration is defined as a tear of the lung parenchyma resulting in the formation of a well-circumscribed opacity or cavity filled with air (pneumatocele), blood (pulmonary hematoma), or both [7]. Multidetector CT is the standard modality for assessing the degree of pulmonary laceration [8]. In blunt trauma, pulmonary laceration is frequently complicated with pneumothorax and hemothorax, often requiring immediate treatment by tube thoracostomy [3, 4]. Most of pulmonary lacerations resolve spontaneously with conservative treatment. The surgical indications for them include intrathoracic hemorrhage of more than 1,000 mL of immediate blood drainage during initial tube thoracostomy or 200 mL/h for 4 consecutive hours, massive air leakage without reexpansion of the lung, and ventilation failure due to airway bleeding [3, 4]. In the present case, the patient did not meet these criteria before the rupture of pulmonary hematoma.

Pulmonary hematoma extending along segmental pulmonary artery in the helium of the lung can rupture into the thoracic cavity. The rapid deceleration occurring in a falling accident produces a shearing force, causing a sudden rotation of the middle lobe of the right lung [9]. In the present case, the mechanism of injury disrupted the hilar structure of the middle lobe, resulting in traumatic pneumothorax and pulmonary hematoma. Subsequent obstruction of the middle lobe bronchus resulting from external compression by an enlarged hematoma in the central portion of the middle lobe reduced the airflow through the leaking fistula. The sudden rupture of pulmonary hematoma into the thoracic cavity probably occurred because of exacerbation of the bleeding tendency in acute DIC following blunt multiple trauma.

VATS has been widely used in the management of acute thoracic trauma [5]. However, the main contraindication to VATS in thoracic trauma patients is hemodynamic instability where an open thoracotomy would be prudent [9, 10]. In this particular case, video-assisted minithoracotomy allowed easier access to the lacerated lesion, enabled good exposure of the entire hemithorax, and provided adequate space for achieving compression hemostasis and for all lobectomy maneuvers. In addition, this minimally invasive approach could contribute to less damage of the chest wall, resulting in faster weaning from mechanical ventilation and reduction of a potential risk of aggravation of acute DIC. Because techniques of video-assisted minithoracotomy have been mainly applied in lobectomy for lung cancer [6], their safety and efficacy for trauma patients with hemorrhagic shock have not been fully evaluated yet. We believe that video-assisted minithoracotomy could be an alternative approach for the treatment of pulmonary lacerations with a massive hemothorax resulting in hemorrhagic shock.

In conclusion, although a massive hemothorax caused by the rupture of a pulmonary hematoma is a rare condition, it should be recognized as a serious potential complication of pulmonary laceration after blunt trauma. Video-assisted minithoracotomy can be helpful in the treatment of this life-threatening condition in hemodynamically unstable patients.

## Abbreviations

CT: Computed tomography
VATS: Video-assisted thoracoscopic surgery
DIC: Disseminated intravascular coagulation.
